# Treatment of Internal Strangulation of the Intestines

**Published:** 1852-08

**Authors:** 


### ECLECTIC DEPARTMENT,

### AND SPIRIT OF THE MEDICAL PERIODICAL PRESS.

---

### *Treatment of Internal Strangulation of the Intestines.*

By R. Robinson, M. R. C. S.

In the treatment of internal strangulation of the bowels, the following indications present themselves. 1. To open the bowels. 2. To subdue inflammatory action. 3. To support the strength. 4. To remove the obstruction.

1. *To open the bowels.*—As constipation is one of the most constant, and earliest symptoms, to relieve the bowels is the earliest indication and thus may be attempted,—*a*, by purgative; *b*, injections; *c*, the warm bath; *d*, nauseating remedies; *e*, bleeding.

*a. Purgatives.*—Great judgment is required in the selection of purgatives, as if they fail they do much mischief. Had I grounds for suspecting intestinal obstructions, I would give one full dose of calomel to liquefy the motions, and follow it up by drachm doses of sulphate of magnesia. If this did not succeed, I would rub croton-oil on the abdomen, or try one drop internally. Failing with these I would give up purgatives altogether.

*b. Injections.*—Purgatives failing to procure action from the bowels, recourse is naturally had to injections, which should, in the first instance, consist of the compound extract of colocynth, dissolved in water; but when a tympanitic state of the bowels occurs, turpentine, or the tincture of assafœtida is to be preferred. So far, however, as my experience goes in these cases, injections avail but little; and I shall hereafter relate a case where seventy-two injections were administered without any apparent beneficial result.

*c. The Warm Bath* is a remedy which it would be very proper to use in the earlier stages; but, as the disease advances, the strength of the patient being much exhausted, would scarcely justify its trial. Fomentations, often useful, and always soothing, should be used in all cases, especially where there is pain.

*d. Nauseating remedies*, in a strong and robust individual, might deserve a trial. Of these, perhaps, the two best are tartarised antimony, and the tobacco injection: but I should not be very sanguine of obtaining success by their means.

*e. Bleeding* may be now and then resorted to in the earlier stages, in the robust and plethoric; but bleeding, carried to any great extent, I consider objectionable, for reasons to be stated hereafter.

2. *To remove inflammation* is a point to which particular attention should be directed; and, perhaps, there is but one thing of more consequence than this, for if it be allowed to go on unchecked, it may destroy the patient; and yet, if too vigorously attacked, (by reference to the cases it will be seen that, in some, it was entirely absent, and, in almost all, limited, and by no means severe,) the patient may sink from other causes. I think, therefore, that general bleeding will seldom be called for on this account; the application of leeches, fomentations, and the administration of small doses of calomel and opium, will be all that is required for removing or controlling peritonitis brought about by this cause.

3. *To support the Strength.*—I am very anxious to lay great stress upon this point, because I do not think it has been sufficiently attended to in practice; and I feel sure that patients have been largely bled, who would have had a better chance, had the vital fluid been less unceremoniously abstracted. If the bleeding be not sufficient to effect its object, either by opening the bowels by its depressing effect, or by removing the peritonitis by its antiphlogistic power, it must, if carried to a great extent, do infinite harm, as it will tend to depress the vital powers, already at a low ebb, and thus take away, I may say, every chance, either from the efforts of nature, or the resources of art. In the absence of fever, and where the stomach would allow of it, I would give, from time to time, barley-water and chicken broth in small quantities. Where there is great restlessness and want of sleep, and where I had failed in the use of purgatives, and determined no longer to administer them, I would certainly advise the administration of opium, as, by so doing, I should hope to tranquillize the nervous system; for nothing tends to exhaust the frame so much as long continued nervous irritability; and cases are on record showing the good that has been done with it occasionally. If I employed opiates, I should prefer solid opium to every other form,—first, as being more likely to be retained by the stomach; and secondly, as possessing a stimulating as well as sedative property, which, I think, would, in these cases, be decidedly advantageous.

4. *To remove the obstruction* is one of the most important and difficult questions connected with this subject, and one upon which much difference of opinion exists. It is clear that this can only be done with certainty by means of an operation; and two cases have lately occurred, in which encouragement has been given to this plan. In one of the cases (Mr. Hilton's,) the bowel was so far liberated, that the intestinal contents passed through the obstructed part. In neither of the cases, however, did the patient long survive. In considering this subject, four points are to be entertained: A, the likelihood of finding and removing the obstruction; B, the place of performing the operation; C, the time at which it should be undertaken; D, the chance of success that may attend the attempt.

A. *The likelihood of finding and removing the obstruction.*—Upon this point there can be no doubt that there is great uncertainty; for although, in both the cases to which I have alluded, the obstruction *was* detected and removed, yet in one (Mr. Hilton's case,) very considerable difficulty was experienced; and in case XVI, had an operation been undertaken, the incision would, in all probability, have been made to the *right* of the umbilicus, as a decided induration was evident there, and not elsewhere, and a pouch immediately above it. It was natural, therefore, to suppose that this was the point of obstruction; whereas, on dissection, this proved to be merely hardened scybala; and the fatal incarceration was situated in the upper and posterior part of the *left* side of the pelvis.

B. *The place of performing the operation.*—Mr. Phillips says: "There are some cases where the seat of obstruction is so clearly indicated, that *no doubt* remains. In such cases, I apprehend, the rule is evident,—the incision should be made as near as is prudent to that point. But supposing the point of obstruction to be only obscurely marked, or indeed not discoverable at all, then I consider the incision should be made on the median line, because an opening in that situation may be found most convenient for liberation, if that be practicable; or, for the establishment of an artificial anus, supposing liberation of the intestine be not accomplished." The case, however, to which I have just alluded, induces me to think the central incision, as recommended by Dr. Crisp, preferable in all cases where the obstruction is seated in the small intestines.

C. *The time at which an operation should be undertaken.*—It is very difficult to lay down any decided rule for this. Mr. Phillips says, that interference by surgical operation is justifiable when three or four days have passed without any relief from the bowels by ordinary means, providing constipation be complete, and fæcal vomiting continue. I scarcely think we are justified in operating so soon; partly because persons have recovered from constipation of twenty-three days' continuance, and partly for reasons which will appear in the sequel.

D. *The chance of success* I regard as a very important consideration before undertaking any operation; and I cannot think that cases of internal intestinal obstruction, even under favorable circumstances, offer much chance of a successful issue from operation.

In the cases to which I have referred, it has been said, that, had an operation been performed earlier, a different result might have been obtained; and, no doubt, in both cases, delay was caused by the unwillingness of parties interested to give their consent. Can that be a matter of wonder, when it is recollected, that if an operation be undertaken, both the patient and surgeon must be prepared to go *all* lengths? The obstruction may *not* be at the part suspected, it may be *some way* from where the operation was commenced, and a *very large abdominal section* may be required to complete the operation; and it may possibly not be completed at all. How can this be undertaken without very considerable risk? How can such an operation be *proposed early?* And how can any better justification be urged for such a proceeding, than that long since advanced by Celsus: "Satius est anceps remedium experiri quam nullum?"

If it be true, that great difficulty attends the finding of these obstructions, and great danger follows the attempt at removing them by the knife, so that we cannot conscientiously recommend it but as a forlorn hope, it is but reasonable to inquire, whether any other expedient can be resorted to for a similar purpose. And this naturally induces me to ask whether nature ever produces a cure, or whether spontaneous relief is ever obtained? Several are on record, where persons with the symptoms I have described, and, to all appearance, sinking from internal obstruction, were suddenly relieved in the bowels, and gradually recovered. A case is alluded to by Mr. Cooper, where this happened. An elderly lady, residing at Norwich, was under the care of Mr. Colman, suffering from constipation of the bowels, having had no evacuation for four days. The usual purgative remedies were prescribed, but without effect; enemata and more drastic cathartics were tried, but still ineffectually; vomiting and immense distension of the abdomen supervened; the symptoms became more and more urgent, and on the twelfth day from her attack she had had no relief from her bowels. Dr. Alderson was then called in, and asked *what purgative* he would recommend; to which he replied—"None; but a large dose of opium." It was given, and *in a few hours* the bowels were freely opened, and the patient recovered.* What was the precise condition of this lady must ever be open to doubt; but I venture to suggest, that this might have been a case where the bowel was strictured by false membrane, that inflammation and ulceration of this band ensued, and that then the gut was liberated; and I feel convinced that, had the patient lived a little longer, it would have entirely given way, and the patient might possibly have recovered. It is this conviction which makes me lay so great stress upon keeping up the strength of the patient; for as newly-formed parts are less organized than those formed originally, there is a hope, if the strength of the constitution be kept up, that the band may give way before the bowel, and the patient's life be saved. It is from this case that I particularly recommend the renewal of the old plan of metallic mercury; I think its use has not been rightly understood. That it will remove an introsusception, or enable a portion of bowel to be drawn out of these bands, I agree with Mr. Hilton, is not at all likely to happen; but that it might in favorable cases (Mr. Hilton's was not one of that sort,) by exciting a pressure upon the bowels, break through a false band, I verily believe; and I am more strengthened in this idea by the good that has occasionally followed its use. I have heard of a case, which I believe was of this kind, where metallic mercury appeared to remove a very obstinate constipation, and the patient recovered; and my friend Mr. Lawrence, of Brighton, has mentioned to me, and kindly allowed me to make public, the two following instances, which are cases in point. In one, a boy, æt. 10, was seized, without any apparent cause, with constipation of the bowels, but with no sign of inflammation. He was bled, leeched, took drastic purgatives, and had seventy-two clysters administered. On the twenty-first day of the disease, no motion having been procured, ℥iij of metallic mercury were swallowed; no effect following, the same quantity was repeated on the twenty-third day, after which he felt great weight and pain in the abdomen, and voided with much forcing, an immense quantity of fæcal matter, and all the mercury, minus ʒss; almost fatal syncope followed, but the boy eventually recovered. The other was a case of similar kind, of shorter duration; it occurred in an elderly lady. All purgatives proving unavailing, two doses of metallic mercury, of ℥iv each were given; several motions (and all the mercury, minus ʒj) followed its exhibition after six hours, but the exhaustion and the depression occasioned, were such as to destroy the patient. For these reasons, I think metallic mercury again worthy of a trial; it *can* do no great harm, and *may* do good. That it will often fail, I have not the least doubt, especially where the obstruction is low down, and has been so great as to ulcerate or destroy the coats of the bowel; but where, on the contrary, the band is thin, and high up in the canal, where the constriction is not so great as seriously to engorge or injure the part constricted, where the system does not sympathize much with the local malady, and where the powers of life remain vigorous, I am not without hope that it may *occasionally* succeed; and if *but one case* should occur in which, from what I have said, a trial of this remedy should again be made with success, I shall consider my observations not altogether out of place, and I hope I may with truth be permitted to say—"Est quiddam prodire tenus si non detur ultra."—*London Jour. of Med.*

